# Human Immunodeficiency Virus Type 1 Nef protein modulates the lipid composition of virions and host cell membrane microdomains

**DOI:** 10.1186/1742-4690-4-70

**Published:** 2007-10-01

**Authors:** Britta Brügger, Ellen Krautkrämer, Nadine Tibroni, Claudia E Munte, Susanne Rauch, Iris Leibrecht, Bärbel Glass, Sebastian Breuer, Matthias Geyer, Hans-Georg Kräusslich, Hans Robert Kalbitzer, Felix T Wieland, Oliver T Fackler

**Affiliations:** 1 Heidelberg University Biochemistry Center (BZH), Heidelberg, Germany; 2 Abteilung Virologie, Universität Heidelberg, Heidelberg, Germany; 3 Institut für Biophysik und Physikalische Biochemie, Universität Regensburg, Regensburg, Germany; 4 Max-Planck-Institut für molekulare Physiologie, Abteilung Physikalische Biochemie, Dortmund, Germany

## Abstract

**Background:**

The Nef protein of Human Immunodeficiency Viruses optimizes viral spread in the infected host by manipulating cellular transport and signal transduction machineries. Nef also boosts the infectivity of HIV particles by an unknown mechanism. Recent studies suggested a correlation between the association of Nef with lipid raft microdomains and its positive effects on virion infectivity. Furthermore, the lipidome analysis of HIV-1 particles revealed a marked enrichment of classical raft lipids and thus identified HIV-1 virions as an example for naturally occurring membrane microdomains. Since Nef modulates the protein composition and function of membrane microdomains we tested here if Nef also has the propensity to alter microdomain lipid composition.

**Results:**

Quantitative mass spectrometric lipidome analysis of highly purified HIV-1 particles revealed that the presence of Nef during virus production from T lymphocytes enforced their raft character via a significant reduction of polyunsaturated phosphatidylcholine species and a specific enrichment of sphingomyelin. In contrast, Nef did not significantly affect virion levels of phosphoglycerolipids or cholesterol. The observed alterations in virion lipid composition were insufficient to mediate Nef's effect on particle infectivity and Nef augmented virion infectivity independently of whether virus entry was targeted to or excluded from membrane microdomains. However, altered lipid compositions similar to those observed in virions were also detected in detergent-resistant membrane preparations of virus producing cells.

**Conclusion:**

Nef alters not only the proteome but also the lipid composition of host cell microdomains. This novel activity represents a previously unrecognized mechanism by which Nef could manipulate HIV-1 target cells to facilitate virus propagation in vivo.

## Background

The Nef protein of Human Immunodeficiency Viruses is a multifunctional protein critical for high virus titers in vivo. Consequently, disease progression in individuals infected with *nef *deficient viruses is at least significantly delayed [1-3]. These effects are thought to mirror independent activities of Nef that prevent immune recognition of virally infected cells and directly boost the replicative potential of HIV [4,5]. To achieve such optimized spread in the infected host, Nef manipulates a variety of transport and signal transduction processes in cells infected by HIV-1. Modulation of cellular transport paths by Nef affects the surface presentation of an increasing number of cell receptors like e.g. CD4, MHC class I and II molecules and chemokine receptors [6-9]. Equally wide spread are Nef effects on host cell signalling, including various alterations of the TCR cascade in T lymphocytes. According to an emerging view Nef can act as an intracellular inducer of TCR distal events in the absence of exogenous stimulation while signalling by exogenous TCR stimulation is tuned down in the presence of the viral protein [10-14]. Finally, during production of progeny virus, Nef augments the intrinsic infectivity of cell-free HIV particles by a factor 5–10 via a poorly characterized mechanism [15-17].

Associated with cellular membranes by virtue of its N-terminal myristoylation and additional membrane targeting motifs, a subpopulation of Nef resides in detergent resistant membrane microdomains (DRMs) or lipid rafts [18-23]. Lipid rafts are defined as highly dynamic microdomains in cellular membranes that are enriched in sphingolipids, cholesterol and raft-targeted proteins. This particular lipid and protein composition is thought to facilitate protein-protein interactions to create microdomains with distinct biological properties. Lipid rafts have been implicated as platforms for central cellular processes such as signal transduction and protein trafficking but are also utilized as preferred sites for entry and egress of a number of viruses, including HIV-1 [24-27]. Originally defined as resistant to extraction with cold detergent, the existence of these membrane microdomains in living cells has been subject to intense debates [28-32]. This controversy stemmed primarily from the lack of both, appropriate live cell imaging techniques to visualize such assemblies and detergent-free biochemical purification protocols. Over the past years, the application of new dyes such as Laurdan and the real-time visualization of protein dynamics during signalling processes have largely corroborated the membrane microdomain concept [33-37]. Moreover, our previous lipidome analysis of highly purified HIV-1 particles provided an example of a biological membrane generated in the absence of detergent that displays a lipid composition with striking similarity to DRMs [38].

Recent studies suggested that DRM incorporation of Nef spatially separates its individual activities in infected cells. The use of mutated Nef proteins that are enriched in DRMs due to an additional palmitoylation signal or that lack a di-lysine motif that facilitates DRM incorporation of the viral protein, respectively, revealed that Nef activities in receptor transport are largely independent of its raft association [19,21]. In contrast, signal transduction properties of Nef such as the association with the activated form of the cellular Pak2 kinase strictly occurs within membrane microdomains, thus providing spatial compartmentalization of individual Nef activities [19,20,39]. This concept is in line with a recent proteomic analysis that revealed significant alterations in the DRM recruitment of TCR machinery by Nef [40]. In contrast, conflicting results exist as to what extent DRM association of Nef determines its ability to enhance virion infectivity. An early report demonstrated that this Nef activity depends on raft integrity of the producer cell and suggested this to reflect the Nef-mediated recruitment of HIV budding structures into DRMs [23]. In line with a role for lipid rafts in Nef-mediated infectivity enhancement, disruption of DRM association of Nef correlates with a loss of infectivity enhancement [19]. However, DRM recruitment of HIV structural proteins was not observed in another study and DRM enrichment of Nef failed to further boost particle infectivity [21].

The biological properties of Nef were thus far largely explained by its interactions with host cell proteins. A few recent reports however suggest that Nef might also affect biosynthesis and transport of select host cell lipid species. These reports primarily focus on cellular cholesterol and suggest a direct interaction of Nef with this sterol as well as the induction of cholesterol biosynthesis genes by Nef [41,42]. Via a mechanism specific to macrophages, Nef was also reported to alter cholesterol efflux by interacting with the ABCA1 transporter [43]. These results raised the possibility that Nef might influence not only the protein but also the overall lipid composition of host cell membranes to optimize virus replication. To test this hypothesis, we compared in this study the impact of Nef on the lipidome of HIV-1 virions and T lymphocytes DRMs.

## Results

### Association of Nef with membrane microdomains is not limiting for its effects on virion infectivity and viral replication

**Figure 1:**
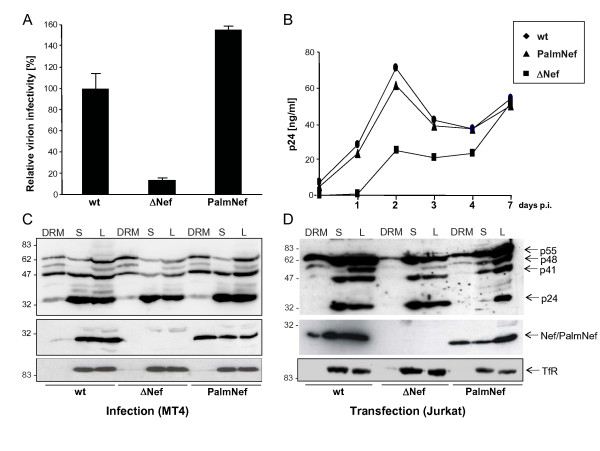
**Nef boosts HIV-1 infectivity and replication without increasing microdomain association of Gag in producer cells**. (A) Single round of replication analysis on TZM cells. TZM cells were infected with 0.5 ng CA of the indicated virus stocks. 36 hours post infection, the cells were fixed, stained for β-galactosidase activity and the number of blue cells was counted. Data represent average values from three independent experiments with triplicate measurements each with the indicated standard error of the mean. Depicted is the relative virion infectivity (number of blue cells per ng CA) with values for HIV-1_NL4-3 _NefSF2 (wt) arbitrarily set to 100%. (B) HIV-1 replication in PBL. HIV replication was measured in 96 well plates on 1 × 10^5 ^PBL per well and 1 ng CA virus input. Freshly isolated, non-activated cells were infected (day -6) for three days and subsequently activated by PHA/IL-2 for three days. Starting from day 0, cells were kept in the presence of IL-2 and cell culture supernatants were collected each day to monitor CA production. CA values represent the average from quadruplicate infections performed in parallel. (C-D) Lipid raft flotation analysis from infected MT-4 (C) or transfected Jurkat T lymphocytes (D). Cell lysates (1% Triton X-100) were separated by Optiprep gradient ultracentrifugation, and eight fractions were collected from the top (fraction 1) to the bottom (fraction 8) of the gradient. The detergent resistant membrane fraction (DRM, fraction 2) and the pooled nonraft (soluble) fractions (S, fractions 7 and 8) were analyzed together with the unfractionated cell lysate (L) by Western Blotting for the distribution of Gag (top), Nef (middle) and TfR (bottom).

We set out to analyze the lipidome of HIV-1 particles produced from MT-4 T lymphocytes in the presence (HIV-1 wt, wt) or absence (HIV-1ΔNef, ΔNef) of Nef. As additional control, we generated a corresponding proviral HIV-1 clone that encodes for a palmitoylated and thus DRM enriched Nef variant [20,21] (PalmNef). Since the effects of Nef on particle infectivity can depend on the nature of the producer cell [44,45], we first analyzed virions produced from infected MT-4 T lymphocytes for their infectivity in a single round of replication on CD4-positive HeLa cells (Fig. 1A). As expected, infection with wt resulted in approx. 7-fold more productively infected cells per ng p24CA virus input than the ΔNef virus. PalmNef was only slightly more efficient (approx. 1.5-fold) than wt Nef in this assay. Similar results were obtained when HIV-1 replication was monitored over several rounds on human primary T lymphocytes: while wt and PalmNef expressing HIV-1 variants replicated with indistinguishable kinetics, spread of HIV-1ΔNef was delayed early post infection (Fig. 1B). Thus, Nef robustly enhances the infectivity of virions produced in MT-4 cells and DRM association of the viral protein is not limiting for this activity.

### Nef does not recruit HIV Gag into detergent resistant microdomains in infected MT-4 T lymphocytes

To address potential reasons for the Nef-mediated increase in virion infectivity in our experimental system, we performed raft flotation experiments with lysates of MT4 T lymphocytes infected with cell free virus stocks (Fig. 1C). Cells were lysed in the presence of cold Tx-100 and, following a standard raft flotation procedure [20], equal volumes DRM (DRM) and soluble (S) fractions as well as total cell lysate (L) were analyzed by Western Blotting. The DRM excluded transferrin receptor (TfR) was used as loding control (bottom panel). Only small amounts of Nef were detected in the DRM fraction and the microdomain association of PalmNef was significantly more pronounced. Pr55^Gag ^precursor, processing intermediates as well as fully processed p24CA were detected with the anti-p24CA antibody (upper panel). The low levels of p24CA in DRM fractions do not reflect a processing defect but rather the lack of membrane targeting after physical separation of CA from MA by protease cleavage. No Nef-mediated enrichment of Pr55^Gag ^in DRMs was detected in infected MT-4 T lymphocytes (ratio of DRM-associated relative to total Gag: wt: 39.4%; ΔNef: 51.4%; PalmNef: 36.1%). These results are in agreement with a study by Sol-Foulon et al. [21] that used HIV-1 infected Jurkat T lymphocytes, however are in conflict with a report on Nef-mediated DRM recruitment of Gag in 293T cells that were transfected with proviral DNA [23]. To assess if this discrepancy stems from the different ways of provirus delivery, we performed the same analysis in Jurkat T lymphocytes transfected with HIV proviral plasmids (Fig. 1D), that expressed higher levels of Gag and Nef than infected MT-4 cells. The presence of Nef resulted in a slightly more pronounced accumulation of Pr55^Gag ^in the DRM fraction under these conditions (ratio of DRM-associated relative to total Gag: wt: 29.8%; ΔNef: 13.4%; PalmNef: 32.3%), suggesting that in Jurkat cells, Nef-mediated DRM recruitment by Nef is only observed upon transfection of proviral DNA. This most likely reflects the unphysiologically high levels of HIV-1 gene products per cell following provirus transfection. Thus, Nef-mediated recruitment of Gag into DRMs does not occur in the context of T lymphocyte infection and is dispensable for Nef's effects on virion infectivity.

### Purification and characterization of HIV-1 virions

**Figure 2:**
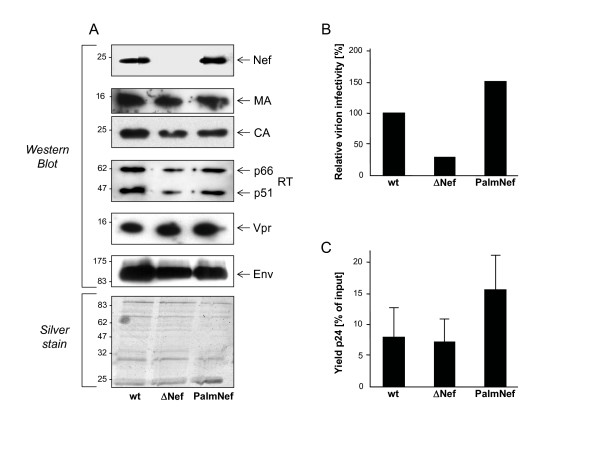
**Characterization of purified HIV-1 particles**. HIV-1 virions were purified from cell culture supernatants (see Materials and Methods for details). (A), Western Blot and silver stain analysis of the indicated virion preparations for major viral particle constituents. (B) Single round of replication analysis on TZM cells with the particle preparations analyzed in A. The assay was performed analogous to that described in Fig. 1A. (C) Relative amounts of total cell culture supernatant p24 recovered after the optiprep procedure. Depicted are average p24 amounts recovered in the virion preparation procedure relative to the total input from four independent purifications with the indicated standard error of the mean.

The above results together with previous reports on the ability of Nef to interfere with cellular cholesterol biosynthesis, homeostasis and transport [41-43] suggested that Nef might increase virion infectivity by altering the composition of the lipid envelope of the particles. Using a previously validated purification scheme that yields particle preparations that are essentially free of vesicle contamination [46], we recently established quantitative lipid mass spectrometry of highly purified HIV particles from infected MT-4 T lymphocytes to determine the lipid composition of HIV virions [38]. We employed this experimental setup to analyze potential differences imprinted by Nef and first assessed the relative incorporation of viral proteins into purified wt, ΔNef and PalmNef particles by Western Blotting (Fig. 2A). No significant difference was detected in the amounts of isolated Gag proteins (MA, CA), viral glycoprotein (Env), viral enzymes (RT) and the virion associated factor Vpr. Comparable amounts of both wt and palmitoylated Nef were also detected, indicating that DRM enrichment does not cause the accumulation of virion-associated Nef under these experimental conditions. Silver staining revealed comparable purity of all preparations analyzed and no significant differences in the virion incorporation of cellular proteins were detected. Notably, the differences in relative infectivity between wt, ΔNef and PalmNef particles seen in cell culture supernatants were preserved following velocity gradient purifications of the particles (Fig. 2B). Furthermore, based on the recovery of viral antigen relative to input amounts prior to the purification, the lack of Nef did not significantly alter the stability of HIV-1 particles (Fig. 2C).

### HIV-1 Nef increases the raft character of virus particles

**Figure 3:**
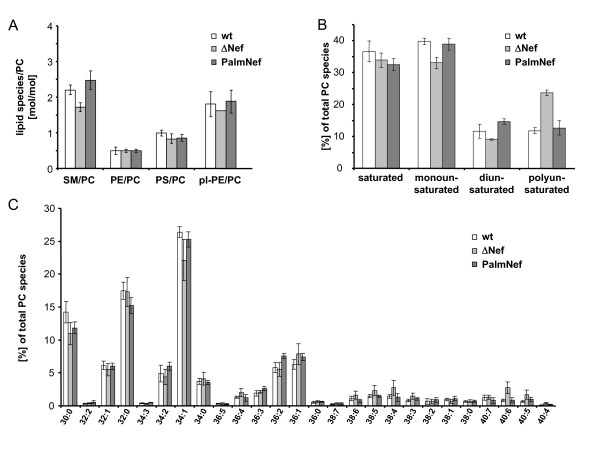
**Lipid analysis of virus particles**. Quantitative lipid analysis was performed as described in Methods. Data are displayed as molar ratio of individual lipid classes to PC. Values present the average from at least three independent experiments with error bars indicating the standard deviation of the mean. (A) PC molecular species distribution given in % of total. (B) Number of species in % of total either containing none, one, or two or more than two double bonds in both fatty acids. (C) Analysis of all PC species. X:Y values on the x-axis denote the total number of C- atoms of both fatty acids (X) and the total number of double bonds (Y), respectively.

We next determined the full lipid composition of the various HIV-1 particle preparations (Fig. 3A). As we reported recently [38], HIV-1 particles display a raft-like lipid composition. In line with the results on DRM incorporation of HIV-1 Gag, the presence or absence of Nef had no global effect on the lipid composition of HIV-1 particles. However, the analysis of individual lipid classes relative to phosphatidylcholine (PC) (that was found to be constant in all particle preparations) revealed a slight but significant enrichment of sphingomyelin (SM) in virions produced in the presence of Nef or PalmNef relative to the ΔNef controls (Fig. 3A) (average SM to PC ratio ΔNef: 1.6, wt: 2.1; p = 0.003 by student's *t*-test). Alterations in cellular SM levels in a similar range have recently been implied to play important roles in Alzheimer's disease [47]. These alterations were specific as Nef had no effect on the virion incorporation of phosphatidylethanolamine (PE), plasmalogen-PE (pl-PE), or phosphatidylserine (PS). Further Nef-specific differences were revealed by the quantitative analysis of PC molecular species (Fig. 3B, C): The presence of Nef and PalmNef during virus production increased the virion amounts of mono- (wt: 39.8% vs. ΔNef: 33.1%, p = 0.01) and di-unsaturated PC species (wt: 11.7% vs. ΔNef: 9.1%, p = 0.0015) while both Nef proteins reduced the virion incorporation of polyunsaturated PC by more than two-fold (wt: 11.8% vs. ΔNef: 23.7%, p = 0.0002). Together these results demonstrate that Nef is not a key determinant for the overall lipid composition of HIV-1 particles but enhances the incorporation of SM and triggers the exclusion of polyunsaturated PC from HIV-1 particles, thereby enhancing their raft microdomain character.

### HIV-1 Nef has no effect on the incorporation of cholesterol into HIV-1 particles

**Figure 4:**
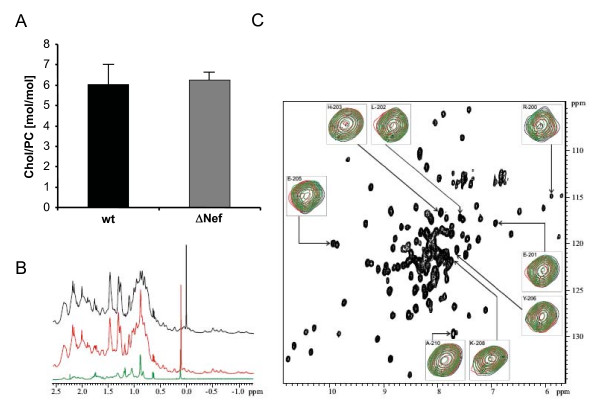
**Nef does not affect the cholesterol content of HIV-1 particles**. (A) Quantitative lipid analysis was performed as described in Methods. Data are displayed as molar ratio of cholesterol to PC. Error bars represent standard deviation of the mean. (B) Selected high-field region of the^1^H spectra of 0.4 mM Nef-SF2 (Δ_1–43_, C210A) in the absence (black) and presence of 0.4 mM water soluble cholesterol (red), in comparison with water soluble cholesterol dissolved in the same buffer as the protein (green).(C) ^1^H,^15^N-TROSY-HSQC spectra of 0.4 mM Nef-SF2 (Δ_1–43_, C210A) highlighting an overlay of the C-terminal residues including the putative cholesterol binding site in the absence (black) and presence of 0.4 mM water soluble cholesterol (red) or 0.4 mM methyl-β-cyclodextrine (green).

Based on the reported direct virion recruitment of cholesterol by Nef, the upregulation of cholesterol biosynthesis in Nef expressing cells, and the importance of virion cholesterol for particle infectivity [41,42,48,49], we specifically addressed the cholesterol content of the isolated HIV-1 particles. As depicted in Fig. 4A, the presence of Nef had no effect on the amounts of cholesterol present in our HIV-1 virion preparations. This prompted us to analyze the proposed direct interaction between Nef and cholesterol in vitro using NMR spectroscopy. This method detects ligand binding by chemical shift perturbation with high sensitivity. The interaction of Nef with cholesterol has been reported to occur via a cholesterol recognition motif Leu_198 _X_1–5 _Tyr_202 _X_1–5 _Lys_204 _[42] at the c-terminus of the well folded core domain of Nef. Thus, cholesterol dissolved in ethanol or cholesterol complexed with methyl-β-cyclodextrine was added in increasing concentrations up to a final ratio of 1:2 to a solution containing the ^13^C/^15^N-labeled core domain structure (residues 44–210) of Nef. Even at the highest concentrations cholesterol specific shifts were neither observed in the ^1^H spectrum (Fig. 4B) nor in the ^1^H/^15^N HSQC spectrum showing the main chain amide signals (Fig. 4C). Thus, no physical interaction between Nef and cholesterol was detected with this highly sensitive in vitro approach.

Besides SM, HIV-1 virions are also highly enriched in the unusual sphingolipid dihydrosphingomyelin (DHSM) and inhibition of sphingolipid synthesis resulted in a 5-fold reduction in virion infectivity [38]. The magnitude of this effect is remarkable close to that Nef exerts on HIV-1 infectivity. However, when we determined the DHSM levels in HIV-1 virions produced in the absence of Nef, no significant change in DHSM virion incorporation was detected (data not shown). Together we conclude that the presence of Nef alters the PC species distribution and SM content of virus particles, while cholesterol and DHSM levels are unaffected.

### Increase of virion SM levels by Nef is insufficient for elevating virion infectivity

**Figure 5:**
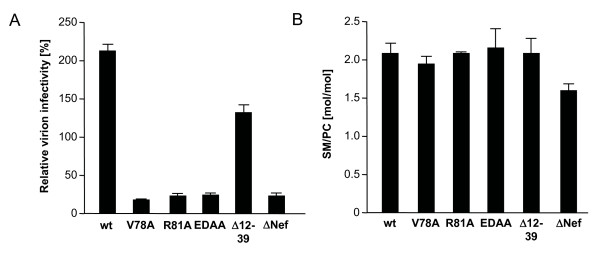
**Elevation of SM/PC ratios is insufficient for Nef-mediated enhancement of virion infectivity**. (A) Single round of infection analysis on TZM cells with cell culture supernatants from MT-4 T lymphocytes infected with the indicated viruses. The assay was performed analogous to that described in Fig. 1A. (B) Quantitative lipid analysis of virion SM/PC ratios. HIV particles purified from the cell culture supernatants analyzed in A were subjected to lipidome analysis. Depicted are the SM to PC ratios.

We next sought to test whether the Nef-induced alterations of viral envelope lipid composition are instrumental for the elevated relative infectivity of virions produced in the presence of Nef. To this end, we analyzed the effect of Nef variants that were previously shown to lack infectivity enhancement potential [50] on the viral lipidome. As shown in Fig. 5A, the V78A, R81A and ED178/179AA variants of Nef failed to augment particle infectivity when compared to ΔNef. A Nef deletion mutant Δ12-39 displayed intermediate activity in this assay. When we analyzed the lipid composition of particles purified from cell culture supernatants used for infection in A, all virion preparations contained SM to PC ratios that were significantly higher than ΔNef particles (Fig. 5B). We therefore conclude that the ability to augment relative SM levels in virus particles is not sufficient to mediate Nef's effects on virion infectivity.

### Enhancement of virion infectivity by Nef is independent of DRM association of the viral entry receptor CD4

**Figure 6:**
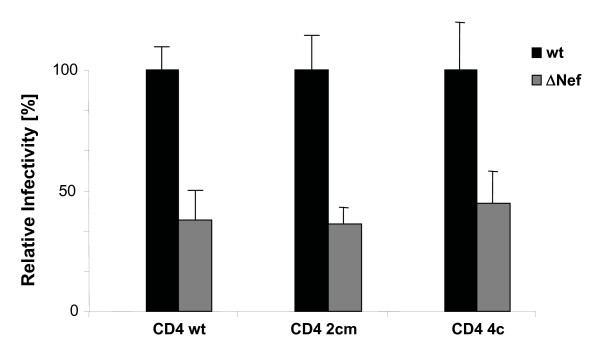
**Nef enhances virion infectivity independently of whether HIV-1 entry occurs via membrane microdomains**. Single round infection analysis on HeLa cells transiently expressing the indicated CD4 variants. Productively infected, p24CA positive cells were quantified 36 hours post infection by flow cytometry. Data represent average values from three independent experiments with the indicated standard error of the mean. Depicted is the relative virion infectivity with values for HIV-1_NL4-3 _NefSF2 (wt) arbitrarily set to 100%.

Entry of HIV-1 into target cells occurs at the plasma membrane and several but not all studies suggested that membrane microdomains represent a preferential local environment for this process [51-56]. While modulation of the virion lipid composition was insufficient to explain the positive effects of the viral protein on particle infectivity, this effect of Nef occurs via a microdomain-dependent mechanism [23]. We therefore reasoned that the increased microdomain character of virions produced in the presence of Nef might specifically increase infection events that occur via lipid raft domains, e.g. by augmenting binding affinity or fusion efficiency of particles at target cell microdomains. To test this hypothesis, we made use of mutations in the primary entry receptor for HIV-1, CD4 that specifically target the receptor to or exclude it from membrane microdomains and tested whether Nef's effect on virion infectivity depends on the DRM localization of CD4. While wt CD4 is distributed approximately equally between DRM and detergent-soluble fractions, mutation of the palmitoylation acceptor in the 2Cm CD4 variant significantly reduced its raft association (approx. 25% in DRM) and additional mutation of the Lck binding motif in the cytoplasmic tail in the 4C CD4 variant almost completely disrupted its DRM association (approx. 5% in DRM) [57]. Wt, 2Cm and 4C CD4 variants were transiently expressed in HeLa cells which were subsequently infected with wt or ΔNef HIV-1. Control experiments confirmed the expected segregation of these CD4 variants between DRM and detergent-soluble fractions as well as comparable cell surface levels for all three proteins (data not shown). The percentage of productively infected cells was determined by flow cytometry of intracellular p24CA at 36 h post infection. Using this experimental set-up, Nef positive virions were approx. 3-fold more infectious than their ΔNef counterparts when wt CD4 was used as entry receptor (Fig. 6, CD4 wt). Of note, comparable differences between the relative infectivity of both viruses were detected when virus entry occurred via raft-excluded or raft enriched CD4 variants (Fig. 6, CD42cm, CD4 4c) and the relative infectivity of wt was comparable irrespective of which CD4 variant was used. Thus, HIV-1 entry does not strictly depend on the raft localization of its primary entry receptor and enhancement of virion infectivity by Nef is independent of whether or not virus entry occurs via raft microdomains.

### Nef modulates the lipid composition of DRMs in infected T lymphocytes

**Figure 7:**
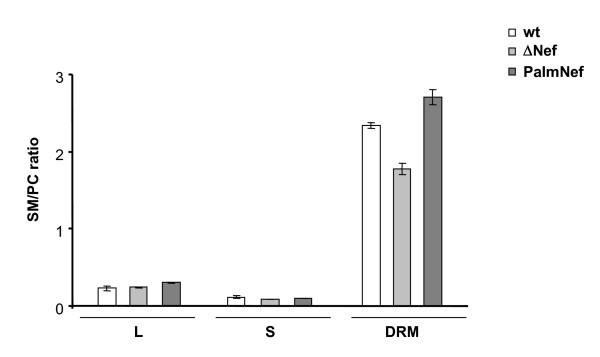
**Nef alters the lipid composition of DRMs in HIV-1 infected T lymphocytes**. DRM flotation analysis was performed from MT4 cells infected with the indicated viruses as described for Fig. 1D and quantitative lipid analysis was performed from L, S and DRM fractions. Depicted are the SM to PC ratios.

We finally sought to address whether Nef selectively changes the incorporation of individual lipid classes into lipid microdomains of the host cell plasma membrane. To this end, we performed a lipid analysis of different fractions of HIV-1 infected MT4 T lymphocytes harvested in parallel to the cell culture supernatants that served as source for infectious virions in the previous analyses (i.e. at a time point where over 90% of all cells are productively infected). Total cell lysates (L) as well as soluble (S) and detergent-resistant (DRM) fractions of flotation gradients were compared regrading their SM to PC ratio (Fig. 7). No significant differences in the SM to PC ratio of the L or S fractions were observed in the absence or presence of Nef. In contrast, DRMs were significantly enriched in SM relative to PC in cells infected with Nef or PalmNef expressing HIV-1 when compared to cells infected with the ΔNef virus. This scenario was remarkably similar to the results obtained with highly purified HIV-1 virions. We conclude that Nef not only emphasizes the microdomain like lipid composition of HIV-1 virions but also alters that of membrane microdomains of virus producing T lymphocytes.

## Discussion

Building on our previous lipidome analysis of highly purified HIV-1 virions, this study tested the hypothesis that Nef affects the lipid composition of HIV-1 particles. Quantitative mass spectrometry indeed revealed that Nef underscores the microdomain character of the viral lipid envelope by increasing virion SM levels and reducing the amounts of polyunsaturated PC in HIV-1 particles. As these effects were also detected in DRM preparations from virus producing cells, the reported modulation of lipid composition of complex membrane bilayers adds a novel mechanism by which Nef can manipulate HIV-1 host cells.

Based on previous reports on the interaction with Nef and the resulting virion incorporation [42] we expected cholesterol to serve as a positive control for Nef effects in our lipidome analysis. Surprisingly however, we failed to detect any appreciable impact of Nef on virion cholesterol levels. These opposing results may reflect different experimental settings in both studies: Zheng et al. measured the uptake of radiolabeled cholesterol while in the present study overall cholesterol levels were quantified by highly sensitive nano-mass spectrometry. In line with our findings, we also failed to detect a physical interaction between Nef and cholesterol in solution by NMR spectroscopy. Such an interaction had been concluded from competition experiments of [17α-methyl-^3^H]-promegestone with water-soluble cholesterol [42]. Using even higher concentrations of up to 800 μM water soluble cholesterol or cholesterol dissolved in ethanol we could not detect a specific interaction of cholesterol with Nef, however. In particular, no chemical shifts were observed for the putative C-terminal cholesterol binding motif **L**_198_HPE**Y**Y**K **in the presence of cholesterol (Fig. 4C). Irrespective of the basis for these differences, our results clearly demonstrate that Nef augments virion infectivity in the absence of elevating cholesterol levels of particles produced from infected T lymphocytes. Our study however does not exclude that Nef affects cholesterol homeostasis e.g. by upregulation of cholesterol biosynthesis genes [41,42], which may not be reflected in bulk virion levels. Additionally, effects of Nef on cholesterol transport were recently reported in macrophages, where the interaction of Nef with the ABCA1 transporter and the resulting reduction of cholesterol efflux were suggested to be instrumental for Nef-mediated enhancement of virion infectivity [43]. While this particular mechanism is not in place in T lymphocytes due to the lack of ABCA1 expression in this cell type, these results emphasize that Nef can affect lipid transport in HIV target cells.

Conflicting previous results caused a controversy as to whether the association of Nef with membrane microdomains contributes to its enhancement of virion infectivity [18,21,23]. We confirm here that in the context of T lymphocyte infection, Nef does not facilitate recruitment of HIV-1 Gag into DRMs and that an experimental increase in Nef's DRM association does not augment its effect on particle infectivity [21]. The results presented also indicate that analysis of DRM incorporation of viral gene products following transfection of proviral DNA, which results in much higher copy numbers of these proteins per cell than following virus infection, should be interpreted with care. Even in the absence of elevated DRM recruitment of Gag, microdomain integrity is a prerequisite for Nef-mediated infectivity enhancement and specific disruption of Nef's microdomain association interferes with this activity [19,23]. Together these results suggest that while microdomain association of Nef is critical for the positive effect of Nef on particle infectivity, this effect is already saturated at the physiological, moderate levels of Nef microdomain incorporation. Consistent with the well accepted model that Nef exerts its effects on virion infectivity during virus production [58], the viral protein augmented particle infectivity independently of whether virus entry occurred via microdomain targeted or excluded entry receptors. We cannot exclude that the microdomain association of the CD4 variants used changed upon engagement by the virus. Nevertheless our results are consistent with the concept that Nef modifies HIV-1 particles during production in a microdomain dependent manner to facilitate early, microdomain independent, post entry steps in the new target cells. Based on the low amounts of Nef present in microdomains, this scenario would be best compatible with a catalytic post entry event that is affected by Nef. In this context Qi and Aiken recently presented an intriguing model that predicts Nef to counteract proteasomal degradation of HIV-1 particles following entry into new target cells [59]. Our analysis of Nef mutants revealed that the observed alterations in particle lipid composition were not sufficient to mediate Nef's effect on virion infectivity. However, as the ratio of e.g. cholesterol and SM critically determines the infectivity of HIV-1 particles [41,42,48,49], these results do not exclude a contribution of the virion lipid composition to this Nef function. Such changes in lipid composition, possibly in conjunction with altered incorporation of host cell proteins, could thus affect post translational modifications and/or conformation of virion determinants that are recognized by the degradation machinery early post entry into a new target cell. This might also involve changes in the membrane microdomain organization of the virions, a parameter that is also critical for optimal particle infectivity [41,42,48,49]. It will be of interest to understand in the future how microdomain association of Nef affects the early post entry fate of HIV virions in the recipient cell.

An important finding of this study is that Nef altered not only the lipid composition of HIV particles but also that of host cell membrane microdomains. Thus, in addition to the well established changes in microdomain protein composition [20,40,60], Nef also affects their lipid microenvironment. The overlap in changes induced by Nef on the lipid composition of bulk host cell microdomains and purified virions implies that the altered particle composition is a direct consequence of the modifications observed in the virus producing cell. The mechanism of this lipid modulatory activity of Nef as well as the molecular determinants for this activity remain unclear. In light of the effects of Nef on lipid transport discussed above, the lack of differences in the lipid composition in total cell lysates suggest effects of Nef on lipid sorting and/or microdomain incorporation as a major determinant of this function. Additionally, Nef may affect microdomain lipid composition by local changes in the turnover of select lipid classes e.g. via modulation of host cell signal transduction pathways and its direct association with lipid kinases such as PI3-K [61]. As lipids potently regulate microdomain activitiy in membrane traffic and signal transduction [62], this mechanism could potentially contribute to a number of Nef's biological properties. The regulatory role of microdomain lipids is particularly prominent during T cell receptor signaling [32,63-65], a process that is altered by Nef in a membrane microdomain dependent manner [19,20,39,40]. Importantly, with Pak2 and PI3 kinases as well as the actin regulator Wasp, these effects of Nef involve its physical or functional association with cellular components whose activity is either subject to regulation by the local lipid microenvironment or causes alterations in the local lipid composition [66-68]. Finally, the altered lipid composition of microdomains in the presence of Nef might have direct consequences of protein incorporation into these domains [69]. Future studies will focus on the functional consequences this novel lipid modulatory activity of Nef exhibits on host cell microdomain function.

## Conclusion

This study describes alterations of the lipidome as a new activity of the HIV pathogenicity factor Nef that might contribute to its multifaceted strategies to manipulate HIV target cells for the optimization of virus spread in the infected host.

## Methods

### Reagents and plasmids

Isogenic proviral constructs expressing various *nef *genes based on the HIV-1 SF2 *nef *sequence in the backbone of the HIV-1 NL4-3 proviral clone were described earlier [50]. The provirus encoding for a Nef protein with palmitoylation site (G3C mutation) [20] was constructed analogously. The complete nucleotide sequences of all *nef *inserts of these proviral clones were verified by sequencing of both DNA strands. The expression plasmids for wt CD4 and the 2Cm and 4C CD4 mutants were kindly provided by Dr. Jin and were described elsewhere [57]. 2Cm lacks the palmitoylation acceptor in the cytoplasmic tail of CD4 while in 4C, the Lck interaction motif is additionally removed. Gag and Nef were detected using the polyclonal rabbit serum CA1 against p24CA [70] and the polyclonal anti-Nef sheep serum Arp444 [71], respectively. Antibodies against Env, RT, Vpr are described elsewhere [50].

### Mass spectrometric lipid analysis of purified HIV particles

HIV-1 virions were purified from cell culture supernatants of infected MT-4 lymphocyte cultures and subjected to quantitative lipid analysis by nano-electrospray ionization tandem mass spectrometry, similarly as described [38].

### Western blotting

For Western blot analysis, samples were boiled in SDS sample buffer, separated by 10% SDS-PAGE and transferred to a nitrocellulose membrane. Protein detection was performed following incubation with appropriate first and secondary antibodies using the super signal pico detection kit (Pierce, Bonn, Germany) according to the manufacturer's instructions.

### HIV replication, single round infectivity, p24 ELISA

The relative infectivity of HIV-1 particles was determined by CA ELISA and a standardized 96 well TZM blue cell assay as described [72]. Briefly, infections were carried out in triplicates with 0.5 ng CA input virus. 36 hours post infection, cells were fixed, stained for β-galactosidase activity and the number of blue cells was determined by microscopy. To analyze the effects of Nef on HIV replication in primary human T lymphocytes [50], peripheral blood mononuclear cells were isolated from healthy donors by Ficoll gradients using Ficoll-Paque Plus (Amersham Biosciences, Uppsala, Sweden). For infection, cells were thawed and kept in bulk cultures in RPMI, 10% FCS at 1 × 10^6 ^cells/ml over night. 1 × 10^5 ^cells/well were then seeded in V-bottom 96 well plates and infected with 1 ng CA virus input per well the following day. 3 days later, cells were washed and stimulated with 2 μg/ml PHA (Sigma) and 20 nM IL-2 (Chiron, Emeryville, CA) for 3 days. After stimulation, the PBL were washed and resuspended in 200 μl of RPMI containing FCS and IL-2. Each day, 100 μl of cell culture supernatant was replaced with fresh medium and amounts of CA in the cell culture supernatant were quantified to monitor virus replication using an in-house p24 ELISA [72].

### NMR spectroscopy

^15^N,^13^C-labelled HIV-1 Nef-SF2 (Δ_1–43_, C210A) of HIV-1 was expressed and purified as described previously [73]. The main-chain amide resonance assignments were taken from the HIV-1 Nef BH10 (Δ_2–39, 159–173_, C206A), whose structure was previously solved by high-resolution NMR spectroscopy [74]. For binding studies 0.4 mM ^15^N,^13^C-labeled Nef (Δ_1–43_, C210A) was used in 5 mM Tris/HCl, pH 8.0, 5 mM DTE, 8% D_2_O, 92% H_2_O, and 0.05 mM DSS (4,4-dimethyl-4-silapentane-sulphonic acid). Cholesterol, water soluble cholesterol (cholesterol in methyl-β-cyclodextrin) and methyl-β-cyclodextrine were purchased from Sigma (C3045, C4951 and C4555, respectively), and used to prepare stock solutions of 40 mM cholesterol in ethanol, 7.5 mM water soluble cholesterol in 5 mM Tris/HCl buffer pH 8.0, and 7.5 mM methyl-β-cyclodextrine in the same buffer.

NMR measurements were performed at 308 K on Bruker Avance 800 MHz spectrometer operating at 800 MHz proton resonance frequency. ^1^H,^15^N-TROSY-HSQC were recorded as described by Pervushin *et al*. [75]. The spectra were recorded with 4 K data points and a spectral width of 14 ppm in the ^1^H dimension, and with 128 data points and a spectral width of 40 ppm in the ^15^N dimension. Proton chemical shifts were referenced to DSS used as internal reference. The ^15^N chemical shifts were indirectly referenced to DSS using the frequency ratio given by Wishart *et al*. [76]. Spectra were acquired and processed with *Topspin 1.3 *(Bruker Biospin, Karlsruhe), and analysed with the program *AUREMOL *[77].

Four identical 0.5 ml samples of 0.4 mM Nef solution were titrated with ethanol, cholesterol in ethanol, cyclodextrine and water soluble cholesterol. The total amount of protein was held constant. ethanol/cholesterol/cyclodextrine was added in increasing amounts up to a molar ratio of protein to ligand of 1 to 2.

## Competing interests

The author(s) declare that they have no competing interests.

## Authors' contributions

BB, EK, NT, CEM, SR, IL, BG and SB performed the experimental work. BB, MG, HGK, HRK, FTW and OTF conceived the experimental strategies and OTF, HRK and BB designed individual experiments. BB and OTF analyzed the data and OTF wrote the manuscript. All authors read and approved the final manuscript.
